# High Performance CMOS Light Detector with Dark Current Suppression in Variable-Temperature Systems

**DOI:** 10.3390/s17010015

**Published:** 2016-12-23

**Authors:** Wen-Sheng Lin, Guo-Ming Sung, Jyun-Long Lin

**Affiliations:** Department of Electrical Engineering, National Taipei University of Technology, Taipei 10608, Taiwan; wensheng.0@gmail.com (W.-S.L.); jdeath000@gmail.com (J.-L.L.)

**Keywords:** light detector, dark diode (DD), photodiode (PD), current amplifier (CA), dark current cancellation, variable-temperature system

## Abstract

This paper presents a dark current suppression technique for a light detector in a variable-temperature system. The light detector architecture comprises a photodiode for sensing the ambient light, a dark current diode for conducting dark current suppression, and a current subtractor that is embedded in the current amplifier with enhanced dark current cancellation. The measured dark current of the proposed light detector is lower than that of the epichlorohydrin photoresistor or cadmium sulphide photoresistor. This is advantageous in variable-temperature systems, especially for those with many infrared light-emitting diodes. Experimental results indicate that the maximum dark current of the proposed current amplifier is approximately 135 nA at 125 °C, a near zero dark current is achieved at temperatures lower than 50 °C, and dark current and temperature exhibit an exponential relation at temperatures higher than 50 °C. The dark current of the proposed light detector is lower than 9.23 nA and the linearity is approximately 1.15 μA/lux at an external resistance *R_SS_* = 10 kΩ and environmental temperatures from 25 °C to 85 °C.

## 1. Introduction

With the ever-increasing demand for eco-design, environmental legislation for electronics is focused on two major requirements. One is the internationalization of the restriction of hazardous substances and the waste of electrical and electronic equipment; the other is a new directive especially for energy using products and the registration, evaluation, authorization, and restriction of chemicals [1]. Lifetime extension is an important eco-design strategy for mitigating the environmental burden of developing new devices. Organic semiconductors increase the lifetime of large-area, low-cost image sensors by several thousand hours, and they monolithically integrate with photonic microsystems [2]. Miniaturized power supply units tend to have a smaller environmental impact at the production stage than traditional electronics do [1]; integrated circuitry can miniaturize power supplies. Colace reported on the first silicon-integrated, 2-D, light-sensitive array fabricated with CMOS technology and readout electronics. The proposed chip includes a light-sensitive array, analog-to-digital converters, dark current cancellation circuitry, and facilities for testing and calibration. It operates as a near-infrared camera [3].

Dark current cancellation is another important eco-design element for photosensors. In reference [4], a hybrid CMOS microfluidic microsystem was proposed for electrochemiluminescence-based biochemical sensing. In the CMOS imager, a two-transistor reset path technique is employed to attenuate the subthreshold leakage current and to reduce the dark current. The imager achieves a low photodiode (PD) dark current of 3.6 nA/cm^2^, but the reset voltage is as high as 2.3 V. However, sub-dark current measurement is completed by subtracting the dark signal frame from the captured frame, and the dark signal frame is stored off chip. Furthermore, a prototype integrated phototransistor-based CMOS active pixel sensor circuit with scintillating material was presented for X-ray imaging. Cancellation of the leakage current using a dummy phototransistor technique was tested and proved efficient [5]. In reference [6], an ultralow dark signal was presented for an embedded active-pixel CMOS image sensor. To achieve in-pixel dark current cancellation, a combined photogate and PD photon-sensing device was developed [6]. Specifically, the dark current was cancelled using a sensing device that was fabricated with a large area. In the current study, dark current cancellation was performed using a current amplifier (CA). A CA is a good photo-detection circuit, which is easily fabricated with CMOS technology. By cancelling the dark current, we can not only enhance the sensitivity of the CA but also reduce the power consumption. The proposed light detector provides a high performance and small chip area.

**Figure 1:**
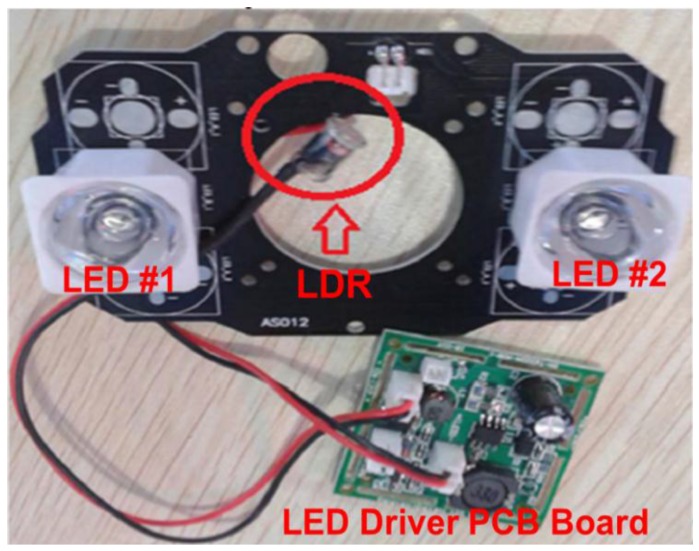
Demonstration board design implemented with the LDR, LED, and LED driver PCB.

A high dynamic range light-to-frequency converter (LFC) chip is proposed with dark current suppression up to 125 °C. By regulating the cathode voltage of the PD and using a replica amplifier, the dark current is reduced. Measurements show that the output frequency is more insensitive to the ambient temperature and process variation. Supply voltage variation is also minimized by implementing a constant delay module [7]. Figure 1 shows a demonstration board design which is implemented with the light-dependent resistor (LDR), the light-emitting diode (LED), and an LED driver printed circuit board (PCB). The typical LED driver with the LDR operates with a large dark current.

In this study, a CA with enhanced dark current cancellation was fabricated using 0.18 µm 1P2M CMOS technology with a chip area of 762 μm × 452 μm, including pads. Simulated and measured results were obtained with white LED light and characterized using a lux meter at a supply voltage *V_CC_* of 3.3 V, an external resistance *R_SS_* of 10 kΩ, and an environmental temperature varying from −40 °C to 125 °C. The rest of this paper is organized as follows. Section 2 elucidates the proposed circuit topology of the light detector. Section 3 presents the simulated and measured results, and in Section 4, conclusions are drawn.

## 2. Proposed Circuit Topology of the Light Detector

**Figure 2:**
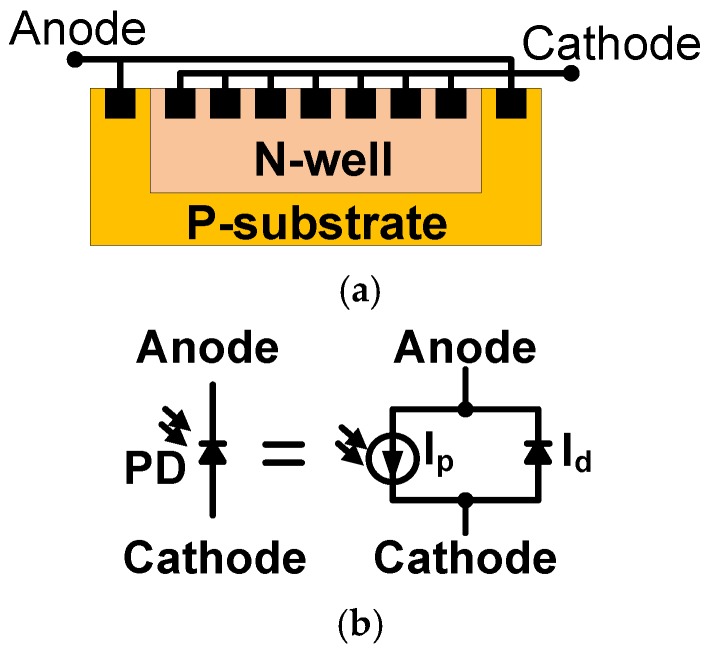
n+/p-substrate PD: (**a**) cross-section; and (**b**) the equivalent circuit.

**Figure 3:**
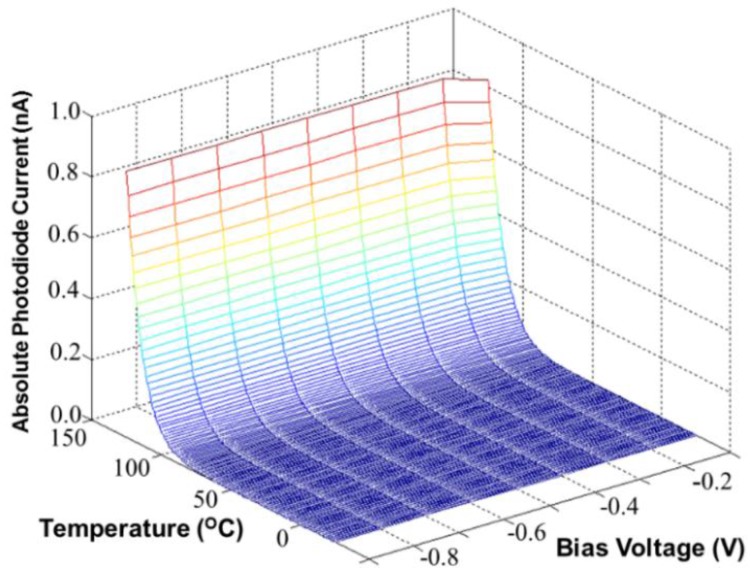
Simulated PD dark currents versus the environmental temperature (°C) and bias voltage (V) in dark conditions.

In this section, we propose a CA with dark current cancellation that is composed of a dark diode (DD) and a photodiode (PD). The DD senses the dark current in dark environment, while the PD senses the photodiode current in electric lighting. Figure 2 presents the schematic cross-section of an n+/p-substrate PD and its equivalent circuit. The PD is built with a p-n junction, which is sensitive to the environmental temperature. Figure 3 shows the simulated PD dark current as a function of environmental temperature (°C) and bias voltage (V) in dark conditions. According to the simulated results, the PD dark current (nA) is highly sensitive to the environmental temperature, but insensitive to the bias voltage.

**Figure 4:**
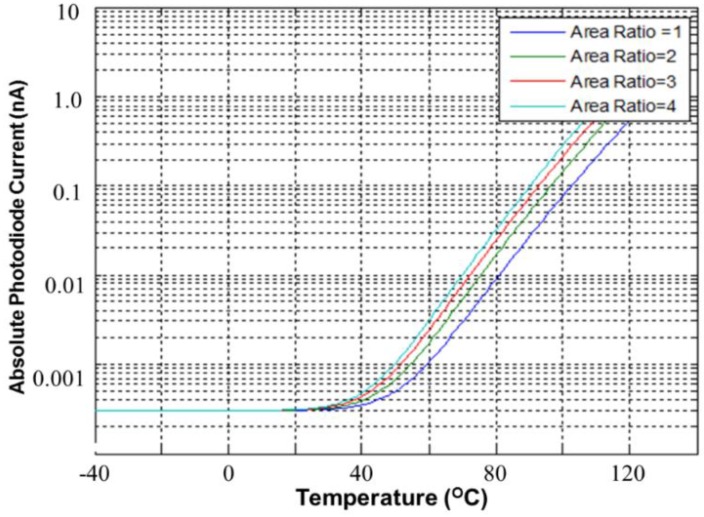
Simulated PD (dark) current versus standardized area ratio in dark conditions.

**Figure 5:**
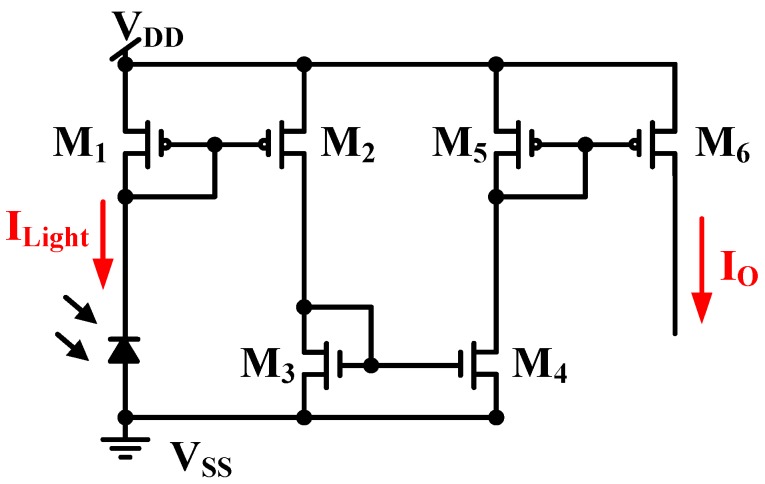
CA without dark current cancellation.

Furthermore, the sensing area of the PD is another important factor. The simulated dark current is approximately proportional to the area ratio, which is defined as the ratio of the PD area to 300 μm × 300 μm. Figure 4 shows the simulated dark current as a function of the environmental temperature (°C) in dark conditions. The smaller the area ratio is, the smaller the dark current is. All simulated dark current levels are settled to 0.3 pA due to imperfect diode model, which is provided from foundry. Figure 5 shows the CA of the PD, which operates without dark current cancellation. The resistance of the PD *R_PD_* is equal to the voltage drop *V_PD_* in the PD divided by its current *I_Light_*. The light current of the PD *I_Light_* is amplified with a current mirror, whose magnification is approximately equal to (*W*_2_/*W*_1_) × (*W*_4_/*W*_3_) × (*W*_6_/*W*_5_) with equal length (*L*) for all MOSFETs. Thus, the output current *I_O_* can be expressed with the total current gain A:

(1) $$\begin{array}{ll} I_{O} & =\frac{\left(W_{2}/L_{2}\right)}{\left(W_{1}/L_{1}\right)}\times \frac{\left(W_{4}/L_{4}\right)}{\left(W_{3}/L_{3}\right)}\times \frac{\left(W_{6}/L_{6}\right)}{\left(W_{5}/L_{5}\right)}\times I_{Light}\\ & =A\times I_{Light} \end{array}$$

where *I_Light_*, also called the photodiode current, includes the illumination current *I_Lux_* and the dark current *I_Dark_*, which flow from *V_DD_* to the ground in a dark environment. The photocurrent *I_Light_* and dark current *I_Dark_* are amplified A times. If the CA operates in a variable-temperature system without dark current cancellation, the dark current causes malfunction. This is the reverse of the characteristic of p-i-n PDs. For a reverse biased diode, the reverse bias current I_R_ can be defined by the following equation with reverse bias voltage *V* [8]:

(2) $$I_{R}=I_{S}\times \exp\left(qV/nkT-1\right)$$

where *I_S_* is the saturation current, *q* is the electronic charge, *kT* is the Boltzman energy, and *n* is a parameter of which the value depends on contributions from both diffusion and surface-generation currents. For a 4H-SiC avalanche photodiode (APD), the dark current is close to the measurement limit at low bias levels. At high bias, the dark current is relatively insensitive to temperature, a characteristic that signifies tunneling. If we assume that the tunneling dark current undergoes avalanche multiplication similar to that of the photocurrent at high bias, the total dark current is expressed by reference [9]:

(3) $$I_{dark}=Gain\left(V,T\right)\times I_{tunneling}$$

where *Gain*(*V*, *T*) is an additional correction factor obtained through photocurrent measurement. The tunneling current *I_tunneling_* is proportional to *A* × *V*^3/2^ with the device area *A* and the effective bias voltage *V* [9]. The objective of this study was to obtain a zero dark current in order to achieve higher photo resistance sensitivity.

**Figure 6:**
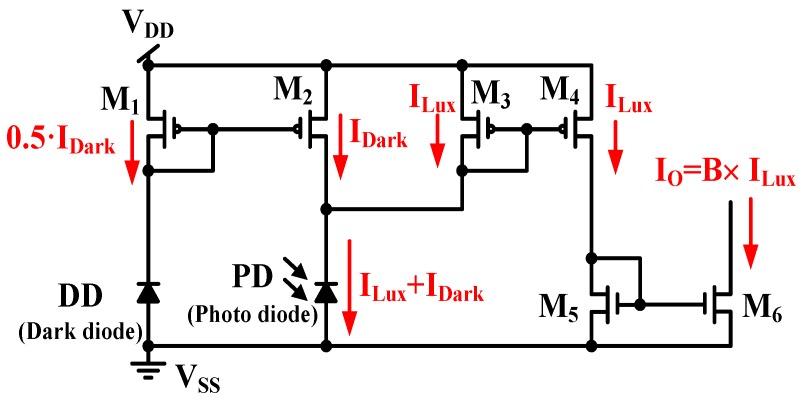
CA with dark current cancellation.

**Figure 7:**
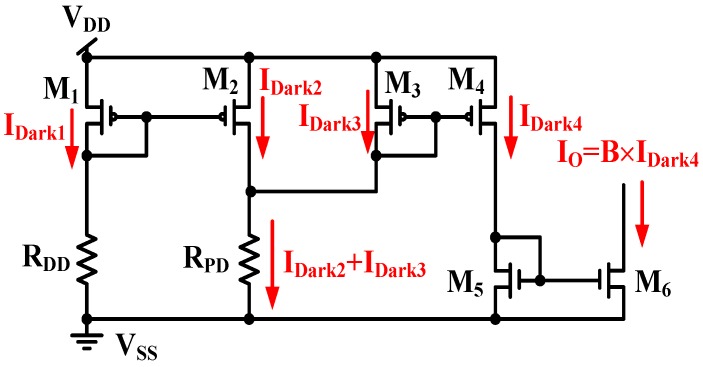
All dark currents of MOSFETs in Figure 6 in dark conditions.

Figure 6 shows a CA with dark current cancellation that is composed of a dark diode (DD) and a PD [10]. The DD senses the dark current *I_Dark_* in dark environments, while the PD senses the photodiode current *I_Light_* in electric lighting. The photodiode size of PD (*A_PD_*) is two times of that of DD (A_DD_); and the MOSFET size of *M*_2_ is two times that of *M*_1_, e.g., *A_PD_* = 2 × *A_DD_* and (*W*/*L*)*_M_*_2_ = 2 × (*W*/*L*)*_M_*_1_. Thus, the drain current *I_D_*_3_ of *M*_3_ is equal to the illumination current *I_Lux_* and the output current I_O_ is expressed by:

(4) $$I_{D3}=I_{Light}-I_{Dark}=I_{Dark}+I_{Lux}-I_{Dark}=I_{Lux}$$

(5) $$I_{O}=\frac{{\left(W/L\right)}_{6}}{{\left(W/L\right)}_{5}}\times I_{Lux}=B\times I_{Lux}$$

where (*W*/*L*)*_n_* is the metric ratio of transistor *M_n_*, and *B* is the current gain. That is, (*W*/*L*)*_M_*_3_ = (*W*/*L*)*_M_*_4_ and (*W*/*L*)*_M_*_6_ = *B* × (*W*/*L*)*_M_*_5_. The output current I_O_ operates without the dark current I_Dark_. That is, the dark current is cancelled in Figure 6. However, it is difficult to achieve a zero illumination current in light application because of the MOSFET mismatch in the CA. When both the DD and PD operate only with dark current, the two equivalent resistors of the DD and PD are expressed as R_DD_ and R_PD_, respectively, and the current symbols, *I_Dark_*_1_, *I_Dark_*_2_, *I_Dark_*_3_, and *I_Dark_*_4_, denote the dark currents of *M*_1_, *M*_2_, *M*_3_, and *M*_4_, respectively, in dark conditions. As shown in Figure 7, the output current I_O_ is achieved:

(6) $$I_{O}=B\times I_{Dark4}$$

where *B* is the current gain displayed in Equation (6) and is approximately 10,000 times. The output current *I_O_* includes the amplified dark current of *M*_4_. This circuit operates with a large dark current.

**Figure 8:**
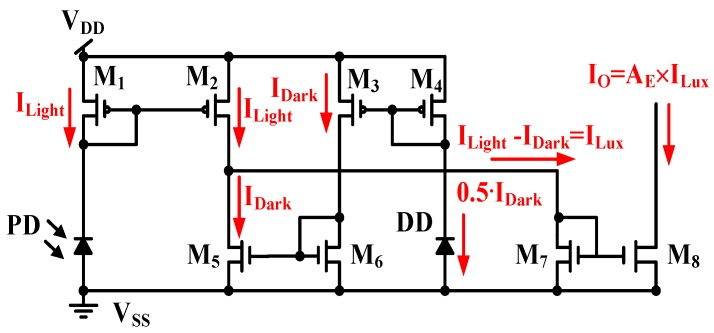
CA with enhanced dark current cancellation.

The dark current in Figure 7 must be reduced. Figure 8 displays a CA with enhanced dark current cancellation. The MOSFET size of *M*_3_ is two times of that of *M*_4_, e.g., (*W*/*L*)*_M_*_3_ = 2 × (*W*/*L*)*_M_*_4_. As shown in Figure 8, current steering is implemented to draw a different current, *I_Light_* − *I_Dark_*, which is equal to the illumination current *I_Lux_*. The output current I_O_ can then be expressed with the total current gain *A_E_*, if (*W*/*L*)*_M_*_1_ = (*W*/*L*)*_M_*_2_, (*W*/*L*)*_M_*_5_ = (*W*/*L*)*_M_*_6_, and (*W*/*L*)*_M_*_8_ = *A_E_* × (*W*/*L*)*_M_*_7_. The output current I_O_ in Figure 8 operates without the dark current.

(7) $$I_{O}=\frac{{\left(W/L\right)}_{8}}{{\left(W/L\right)}_{7}}\times \left(I_{Light}-I_{Dark}\right)=A_{E}\times I_{Lux}$$

**Figure 9:**
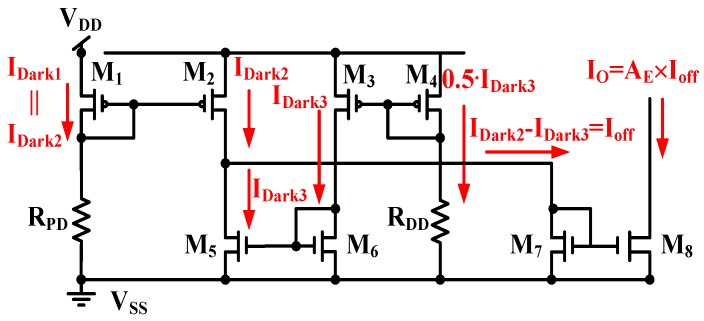
Dark currents of all MOSFETs in the proposed CA with enhanced dark current cancellation.

When both the DD and PD operate only with dark currents, the two equivalent resistors of the DD and PD are expressed as R_DD_ and R_PD_, and the current symbols, *I_Dark_*_1_, *I_Dark_*_2_, and *I_Dark_*_3_, denote the dark currents of *M*_1_, *M*_2_, and *M*_3_, respectively. As shown in Figure 9, the output current I_O_ is obtained.

(8) $$I_{O}=A_{E}\times \left(I_{Dark2}-I_{Dark3}\right)=A_{E}\times I_{off}$$

where *A_E_* is the current gain, as in Equation (7). The output current I_O_ can be reduced to approximately zero if the dark current of *M*_2_ is approximately equal to that of *M*_3_ (*I_Dark_*_2_ ≈ *I_Dark_*_3_). The proposed circuit can also suppress the dark current induced by the environmental temperature, as described in Equation (3).

## 3. Simulated and Measured Results

**Figure 10:**
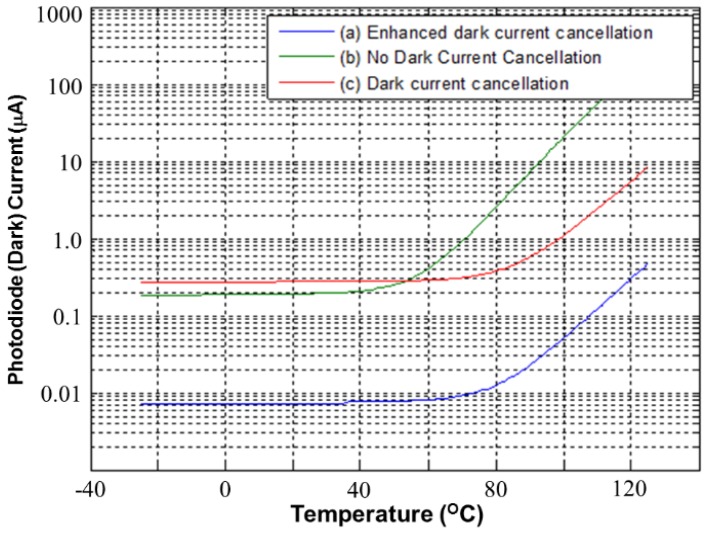
Simulated PD dark currents versus temperatures for various CAs. (**a**) Proposed CA with enhanced dark current cancellation; (**b**) the CA without dark current cancellation; and (**c**) the CA with dark current cancellation.

Figure 10 presents the simulated PD dark current versus temperature for CAs. Three types of CA were compared. First, the proposed CA with enhanced dark current cancellation is indicated by Figure 10a. Next, a CA without dark current cancellation is denoted by Figure 10b. Finally, a CA with dark current cancellation is denoted by Figure 10c. According to the simulated results, the proposed CA with enhanced dark current cancellation operates with the lowest dark current. This means that the dark current can be cancelled perfectly with the proposed CA. The CA without dark current cancellation, which is denoted by Figure 10b, operates with a larger dark current. Note that the dark current level of curve Figure 10c is higher than that of Figure 10b below approximately 50 °C, because the magnification of curve Figure 10c is higher than that of curve Figure 10b.

**Figure 11:**
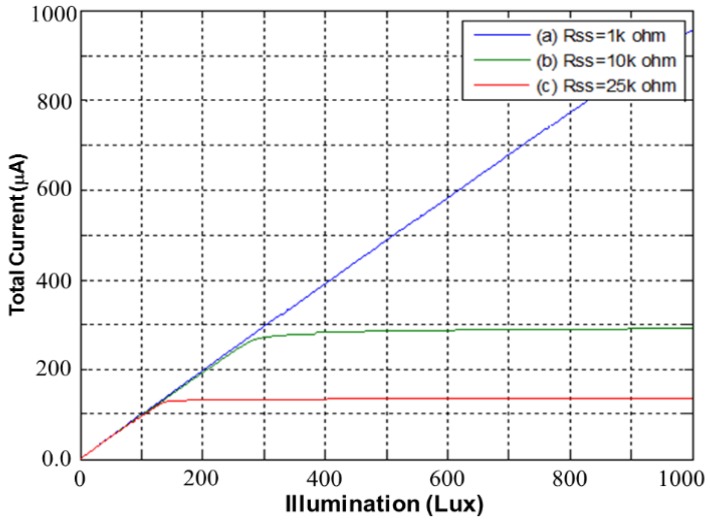
Simulated total currents versus illumination (lux) for the proposed CMOS light detector.

**Figure 12:**
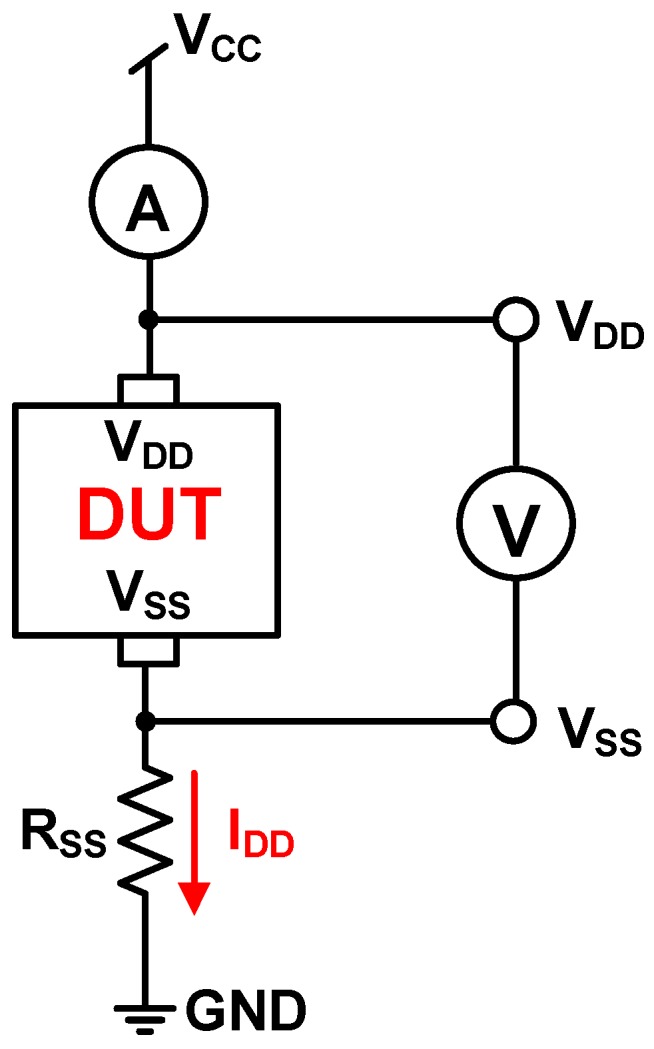
Setup of the proposed CA for measurement.

Figure 11 shows the simulated total current I_DD_ of the proposed CA as a function of illumination at three external resistances, namely *R_SS_* = 1 kΩ, *R_SS_* = 10 kΩ, and *R_SS_* = 25 kΩ. As shown in Figure 11, a lower *R_SS_* expands the illumination detection range. Two higher resistances, *R_SS_* = 10 kΩ and *R_SS_* = 25 kΩ, sharply force the proposed CA into saturation at 280 lux and 140 lux, respectively. Thus, for wide application, selecting a low *R_SS_* is beneficial. Figure 12 shows the setup of the proposed CA that was used for measurement, with a current meter (A), voltage meter (V), and the design under test (DUT) chip. The external resistance *R_SS_* was used to measure the total current I_DD_, which was varied by applying illumination (lux) with white LED light.

Table 1 and Table 2 summarize the simulated and measured dark current *I_Dark_* and total current I_DD_ of the proposed CA with enhanced dark current cancellation at the PD area of 300 μm × 300 μm and a PD to DD area ratio of 2 times. Table 1 indicates that the measured dark currents are smaller than those of the simulations. This is because the PD model was always adopted in the simulations, but is not proven in the silicon process. However, the measured total currents are larger than those of the simulations. The total light current is approximately linear as a function of illumination in the simulation and the measurements in Table 2.

**Figure 13:**
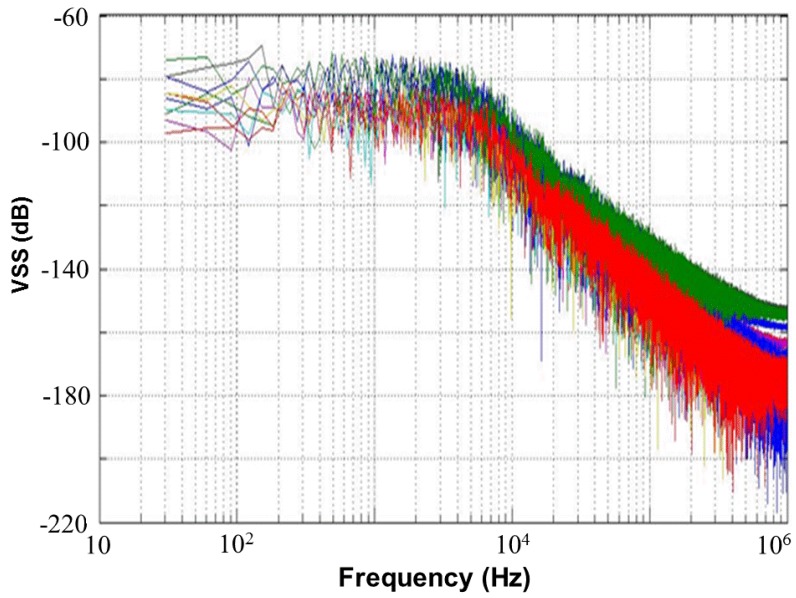
Frequency response of simulated 10 Monte Carlo samples with random noises at node *V_SS_* of Figure 12 for proposed light detector, with *R_SS_* = 1 kΩ.

**Figure 14:**
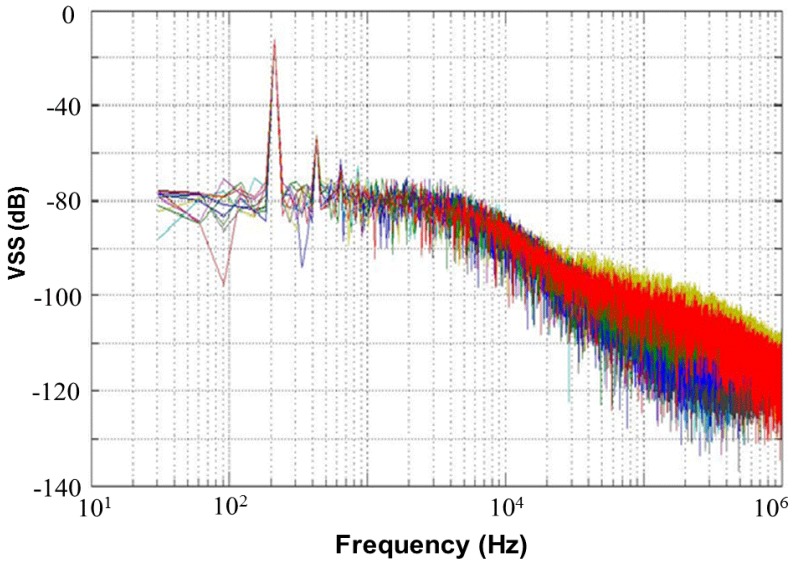
Frequency response of simulated 10 Monte Carlo samples with random noises and a 210 Hz sine wave at node *V_SS_* of Figure 12 for the proposed light detector, with *R_SS_* = 1 kΩ.

Figure 13 shows a 65,536-point FFT simulation with a noise level of 80 dB and an external resistor *R_SS_* of 1 kΩ for the proposed CMOS light detector. This figure presents a simulated frequency response of 10 Monte Carlo samples with random noises at node *V_SS_*, which is shown in Figure 12. If a photodiode current signal, which is a superposition of a 250 lux (peak-to-peak) noiseless sine wave with a frequency of 210 Hz and a DC 500 lux, is fed to the proposed CMOS light detector, a plot of the frequency response of the simulated 10 Monte Carlo samples with random noises is made in Figure 14 at node *V_SS_* for the proposed light detector. The current signal with frequency of 210 Hz performs without any random noises. Table 3 shows the simulated total harmonic distortions (THDs), signal-to-noise ratios (SNRs), and signal-to-noise+distortion ratios (SNDRs) of the 10 Monte Carlo samples with random noises at node *V_SS_* of Figure 14 for the proposed light detector. As shown in Table 3, those simulated results of THD, SNR, and SNDR are roughly consistent. The Monte-Carlo approach can capture very nonlinear noise behaviors.

**Figure 15:**
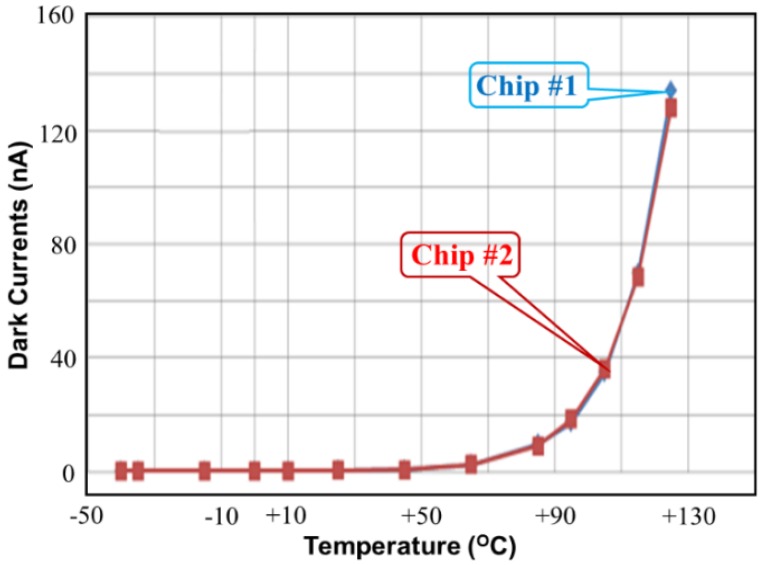
Measured dark currents versus temperatures for the proposed CAs with enhanced dark current cancellation.

Figure 15 shows the measured dark current versus temperature for the proposed CA with enhanced dark current cancellation. The maximum dark current is approximately 135 nA at 125 °C. This measurement proves that the proposed CA performs with low dark current as a function of the environmental temperature. According to the measurements in Figure 15, the device performs with zero dark current at temperatures lower than 50 °C and exhibits an exponential relation at temperatures higher than 50 °C. Notice that two measured dark currents are consistent in Figure 15, although chip #1 and chip #2 perform with different maximum dark currents at 125 °C.

Table 4 summarizes the measured dark currents versus temperature of the proposed CAs, Chap #1 and Chip #2. Table 5 summarizes the measured output currents with respect to the illumination of the proposed CAs. According to the measured output currents in Table 5, the proposed CA performs with good linearity and wide illumination range.

**Figure 16:**
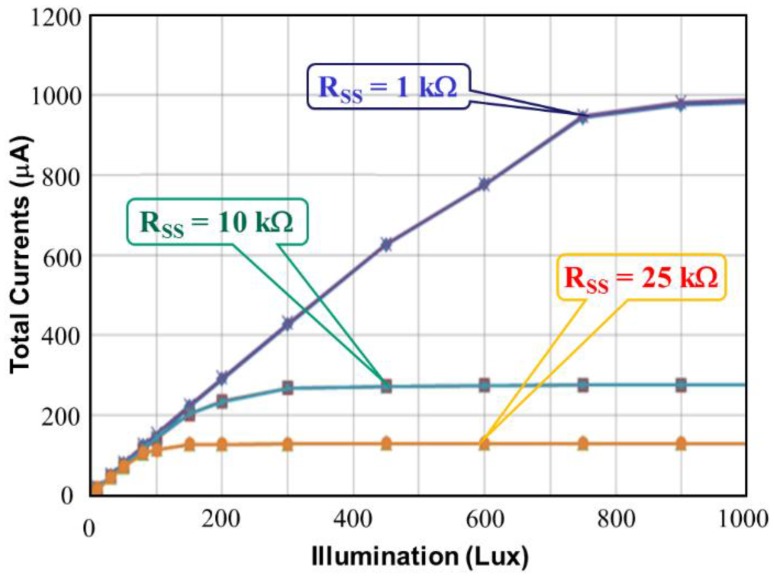
Measured total currents versus illumination (lux) for the proposed CMOS light detector.

**Figure 17:**
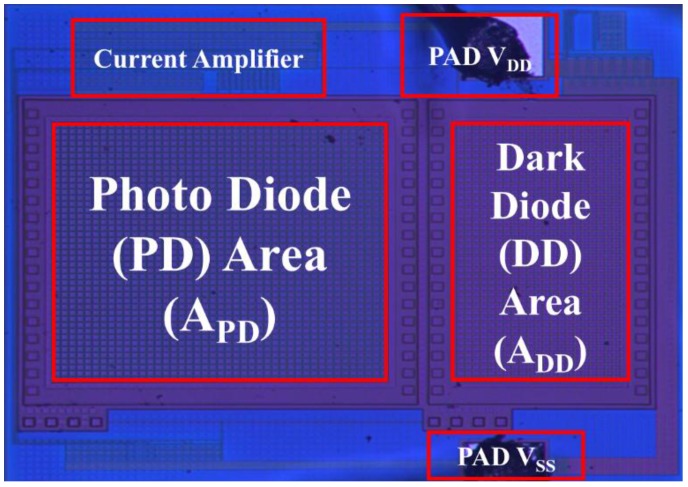
Microphotograph of the proposed CMOS light detector.

Figure 16 shows the total current as a function of illumination (lux) for the proposed CMOS light detector with dark current suppression at room temperature. The larger *R_SS_* is, the lower the total current. As shown in Figure 16, two curves with *R_SS_* = 10 kΩ and *R_SS_* = 25 kΩ indicate saturation at low illumination, but the curve with *R_SS_* = 1 kΩ does not display saturation until 1000 lux. The measured total currents *I_DD_* match those in Figure 11. Figure 17 shows the microphotograph of the proposed CMOS light detector with dark current suppression in a variable-temperature system. Note that the PD area *A_PD_* is twice as large as the DD area *A_DD_*. This arrangement not only lowers the dark current by contracting A_DD_ but also enhances the luminous efficiency by enlarging *A_PD_*.

## 4. Conclusions

This study presents a current steering method for drawing the current difference and lowering the dark current that is applied to the light detector in a variable-temperature system. The maximum dark current of the proposed CA is approximately 135 nA at 125 °C; and that the light detector proposed in this study performs with a low dark current as a function of the environmental temperature. Furthermore, the device obtains a zero dark current at temperatures lower than 50 °C and exhibits an exponential relation at temperatures higher than 50 °C. In addition, the larger *R_SS_* is, the lower the total current is. Two curves with *R_SS_* = 10 kΩ and *R_SS_* = 25 kΩ indicate saturation at low illumination, but the curve with *R_SS_* = 1 kΩ does not display saturation until 700 lux. Low external resistance *Rss* results in a wide detected range of illumination (lux). The PD area A_PD_ is twice as large as the DD area *A_DD_*. This design not only lowers the dark current by contracting A_DD_ but also enhances the luminous efficiency by enlarging *A_PD_*. The chip area of the light detector is 762 μm × 452 μm, including pads. According to the measured results, the proposed CA successfully eliminates dark current from 50 °C to 125 °C.

## Figures and Tables

**Table 1:** Simulated and measured dark currents versus temperature for the proposed CA with enhanced dark current cancellation.

Temperatures (°C)	Simulations	Measurements
25	13.6 nA	1.24 nA
85	21.6 nA	9.23 nA
105	77.0 nA	36.4 nA

**Table 2:** Simulated and measured total currents versus illumination (lux) of the proposed CA with enhanced dark current cancellation.

Illuminations (lux)	Simulations	Measurements
10	8.78 μA	18.2 μA
100	99.3 μA	145.0 μA
200	194.0 μA	236.0 μA

**Table 3:** Simulated THDs, SNRs, and SNDRs of the 10 Monte Carlo samples with random noises at node *V_SS_* of Figure 14 for the proposed light detector.

Samples	1	2	3	4	5	6	7	8	9	10
THD (dB)	−39.84	−39.82	−39.84	−40.41	−39.85	−39.75	−40.27	−40.52	−40.06	−40.75
SNR(dB)	52.60	51.16	50.99	50.15	51.86	52.41	50.39	50.05	51.43	52.64
SNDR(dB)	39.61	39.51	39.52	39.97	39.58	39.52	39.87	40.07	39.76	40.48

**Table 4:** Measured dark currents versus temperature of the proposed CAs, Chip #1 and Chip #2.

Temperatures (°C)	Chip #1	Chip #2
25	1.39 nA	1.24 nA
85	9.7 nA	9.23 nA
105	35 nA	36.4 nA

**Table 5:** Measured output currents with respect to illumination with *R_SS_* of 1 kΩ for the proposed CAs.

Illumination (lux)	Chip #1	Chip #2
10	17.2 μA	18.2 μA
100	142 μA	145 μA
200	237 μA	236 μA
300	426 μA	430 μA
600	776 μA	776 μA
750	943 μA	947 μA
900	975 μA	980 μA
